# No pharmacodynamic (PD) and pharmacokinetic (PK) interaction of riociguat (BAY 63-2521) and aspirin

**DOI:** 10.1186/1471-2210-11-S1-P25

**Published:** 2011-08-01

**Authors:** Reiner Frey, Wolfgang Mück, Sigrun Unger, Michael Reber, Jörn Krätzschmar, Corina Becker, Georg Wensing

**Affiliations:** 1 Clinical Pharmacology, Bayer HealthCare AG, Pharma Research Center, Wuppertal, Germany; 2 Global Biostatistics, Bayer HealthCare AG, Pharma Research Center, Wuppertal, Germany

## Objectives

Riociguat, an oral soluble guanylate cyclase (sGC) stimulator, is a new candidate for treatment of pulmonary hypertension (PH). Riociguat increases cGMP production through a novel dual mode of action: direct NO-independent stimulation of sGC and increasing sensitivity of sGC to low levels of NO. Another sGC stimulator, BAY 41-2272, has shown anti-platelet activity in animal models, as have BAY 41-2272 and riociguat in washed human platelets, although bleeding has not been noted as an adverse event (AE) in riociguat clinical studies [1,2]. As riociguat and aspirin are likely to be used together in PH, it was of interest to investigate potential PD and PK interactions.

## Methods

In this randomized, open-label, crossover study, participants took 2.5 mg/day riociguat, two morning doses of 500 mg aspirin, or both drugs concomitantly.

## Results

Eighteen healthy men were enrolled. Six of 17 participants in the safety evaluation reported ≥1 treatment-emergent AE. All AEs were mild except 1 case of moderate headache following riociguat administration. Fifteen participants were valid for PD/PK analysis. Riociguat PK values were independent of aspirin coadministration. One hour after coadministration of riociguat and aspirin, the mean increase in fraction unbound was 19% for riociguat and 24% for its metabolite M-1 (BAY 60-4552) indicating mild displacement by salicylic acid, the main aspirin metabolite. Effects of aspirin on bleeding time, platelet aggregation and plasma thromboxane B_2_ were not affected by concomitant riociguat. Riociguat alone had no effect on PD variables.

## Conclusion

Riociguat demonstrated no clinically relevant PD or PK interaction with aspirin. Coadministration of riociguat and aspirin does not require dose adjustment. Phase 3 randomized controlled trials are investigating riociguat in chronic thromboembolic pulmonary hypertension or pulmonary arterial hypertension.
